# Mesenchymal Stem Cells Promote Mammosphere Formation and Decrease E-Cadherin in Normal and Malignant Breast Cells

**DOI:** 10.1371/journal.pone.0012180

**Published:** 2010-08-16

**Authors:** Ann H. Klopp, Lara Lacerda, Anshul Gupta, Bisrat G. Debeb, Travis Solley, Li Li, Erika Spaeth, Wei Xu, Xiaomei Zhang, Michael T. Lewis, James M. Reuben, Savitri Krishnamurthy, Mauro Ferrari, Rogério Gaspar, Thomas A. Buchholz, Massimo Cristofanilli, Frank Marini, Michael Andreeff, Wendy A. Woodward

**Affiliations:** 1 Department of Radiation Oncology, The University of Texas MD Anderson Cancer Center, Houston, Texas, United States of America; 2 iMed.UL, Faculdade de Farmácia, Universidade de Lisboa, Lisbon, Portugal; 3 Department of NanoMedicine and Biomedical Engineering, The University of Texas Health Science Center at Houston, Houston, Texas, United States of America; 4 Department of Stem Cell Transplant and Cellular Therapy, The University of Texas MD Anderson Cancer Center, Houston, Texas, United States of America; 5 Lester and Sue Smith Breast Center, Baylor College of Medicine, Houston, Texas, United States of America; 6 Department of Hematopathology, The University of Texas MD Anderson Cancer Center, Houston, Texas, United States of America; 7 Department of Pathology, The University of Texas MD Anderson Cancer Center, Houston, Texas, United States of America; 8 Breast Medical Oncology, The University of Texas MD Anderson Cancer Center, Houston, Texas, United States of America; Baylor College of Medicine, United States of America

## Abstract

**Introduction:**

Normal and malignant breast tissue contains a rare population of multi-potent cells with the capacity to self-renew, referred to as stem cells, or tumor initiating cells (TIC). These cells can be enriched by growth as “mammospheres” in three-dimensional cultures.

**Objective:**

We tested the hypothesis that human bone-marrow derived mesenchymal stem cells (MSC), which are known to support tumor growth and metastasis, increase mammosphere formation.

**Results:**

We found that MSC increased human mammary epithelial cell (HMEC) mammosphere formation in a dose-dependent manner. A similar increase in sphere formation was seen in human inflammatory (SUM149) and non-inflammatory breast cancer cell lines (MCF-7) but not in primary inflammatory breast cancer cells (MDA-IBC-3). We determined that increased mammosphere formation can be mediated by secreted factors as MSC conditioned media from MSC spheroids significantly increased HMEC, MCF-7 and SUM149 mammosphere formation by 6.4 to 21-fold. Mammospheres grown in MSC conditioned media had lower levels of the cell adhesion protein, E-cadherin, and increased expression of N-cadherin in SUM149 and HMEC cells, characteristic of a pro-invasive mesenchymal phenotype. Co-injection with MSC *in vivo* resulted in a reduced latency time to develop detectable MCF-7 and MDA-IBC-3 tumors and increased the growth of MDA-IBC-3 tumors. Furthermore, E-cadherin expression was decreased in MDA-IBC-3 xenografts with co-injection of MSC.

**Conclusions:**

MSC increase the efficiency of primary mammosphere formation in normal and malignant breast cells and decrease E-cadherin expression, a biologic event associated with breast cancer progression and resistance to therapy.

## Introduction

Tumors, like normal tissues, are composed of a heterogenous population of cells with variable capacity for self-renewal. Multipotent tumor cells with the capacity to self-renew and recapitulate the tumors from which they were derived following transplantation into immunocompromised mice are referred to as tumor initiating cells (TIC) or cancer stem cells. TIC can be characterized by specific cell surface marker expression patterns, such as lin^−^/CD44^+^/CD24^−^ or ALDH1 expression [1], [2]. Breast TIC can also be enriched by growth as spheres in anchorage-independent, growth factor enriched, serum-free conditions, referred to as mammospheres [3], [4]. Mammospheres formed from normal human mammary epithelial cells have a higher number of mammary stem cells, which can a form a functional mouse mammary gland *de novo* [5]. Similarly, tumors grown as mammospheres (also known as tumorspheres) are enriched with stem cells markers, lin^−^/CD44^+^/CD24^−^ and ALDH1, and have increased capacity for tumor initiation in xenograft models [6].

We hypothesized that TIC may respond to microenviromental signals which effect signaling and promote their survival. TIC would then resemble normal tissue stem cells in this regard which are dependent on their microenvironment or niche for maintenance of survival factors and suppression of proliferation signals [7]. One candidate cell type within the tumor microenvironment to interact with TIC is the mesenchymal stem cell (MSC). MSC, which are found in the bone-marrow and other tissues, exhibit a marked tropism for tumors and increase tumor metastasis [8], [9].

We analyzed the effect of MSC on mammosphere formation as a surrogate marker for TIC and report that MSC increase primary sphere formation from human mammary epithelial cells (HMEC) and from E-cadherin expressing breast cancer cell lines, MCF-7, SUM149, and a novel inflammatory line MDA-IBC-3. MSC modulated cadherin expression *in vitro* and *in vivo*. This work suggests that MSC secrete factors which promote mammosphere formation which may represent a novel mechanism by which MSC impact breast cancer progression.

## Materials and Methods

### **Cell culture**

MCF-7 breast cancer cells were obtained from American Type Culture Collection (Manassas, VA, USA) and were cultured in modified Eagle medium (MEM) supplemented with 10% heat-inactivated fetal calf serum, 0.1 mM nonessential amino acids and 1 mM sodium pyruvate, 5 µg/ml insulin, 1 µg/ml hydrocortisone and antibiotic-antimycotic. SUM149 cells were obtained from Dr Stephen Ethier (Kramanos Institute, MI, USA) and are commercially available (Asterand, Detroit, MI) and were cultured in Ham's F-12 media supplemented with 10% fetal bovine serum (FBS), 1 µg/ml hydrocortisone, 5 µg/ml insulin and antibiotic-antimycotic. HMEC were kindly provided by Dr Mani (MD Anderson Cancer Center, Houston, USA) and were cultured in MEGM:DMEM-F12 (1∶1) supplemented with insulin, hEGF, hydrocortisone and penstrep. CD16 cells, a differentiated fibroblast cell line obtained from human embryonic lung, were obtained from American Type Culture Collection (Manassas, VA, USA). CD16 fibroblasts were cultured in MEM with Earl Salts and L-glutamine with 10% FBS and antibiotic.

The MDA-IBC-3 cell line was generated from primary human breast cancer cells isolated from pleural effusion fluid obtained on a clinical protocol approved by the institutional review board from a patient with inflammatory breast cancer (IBC). Between October 2006 and September 2009, 29 patients with metastatic breast cancer and symptomatic pleural effusion at The University of Texas M. D. Anderson Cancer Center underwent thoracentesis and provided written informed consent for the use of residual pleural fluid after pathologic assessment for research at The University of Texas M. D. Anderson Cancer Center. The fluid was specifically cultured in accordance with a separate protocol detailing the use and de-identification of this material reviewed and approved by the institutional review board (IRB). Tumor cells were selected by serial transplant into the cleared mammary fat pads of SCID/Beige immunocompromised mice and the resulting tumor tissue passaged in monolayer and 3D culture for the experiments described herein. Characterization and short tandem repeat analysis are provided in the supplemental data (Supplemental Table S1). MDA-IBC-3 cells were cultured in Ham's F12 with 10% FBS and 5 ml Insulin/L with 100 µg/L Hydrocortisone and antibiotic.

Human MSC were isolated from the bone marrow of normal individuals undergoing bone marrow harvest for allogeneic bone marrow transplantation following informed consent, according to institutional guidelines under the approved protocol, as described previously [8]. Briefly, mononuclear cells were separated by centrifugation over a Ficoll-Hypaque gradient (Sigma-Aldrich), suspended in α-minimum essential medium (α-MEM) containing 20% FBS, L-glutamine and penicillin–streptomycin, and plated on 180 cm^2^ dishes. After 3 days, non-adherent cells were removed by washing with phosphate-buffered saline (PBS), and monolayers of adherent cells were cultured until they reached confluence. Cells were then trypsinized (0.25% trypsin with 0.1% EDTA) and subcultured at densities of 5,000–6,000 cells/cm^2^. Cell passages 3–10 were used for the experiments. Characterization of MSC used is provided in the supplemental data (Supplemental Figure S1). Human omental derived adipose stromal cells were isolated from human omentum under an IRB approved protocol according to published protocols for adipose stromal cell isolation [10]. Omentum was derived from a patient with endometrial cancer without omental metastasis.

### Fluorescent marker labeling

Cell lines were labeled with the green fluorescent protein (GFP) using a lentiviral vector, pFUGW [11] that was generously provided by Dr. Baltimore (Caltech). The titers of virus stocks were determined by calculating the percentage of GFP-positive (GFP^+^) 293T cells transduced with serially diluted virus suspensions. For transduction, the MCF-7 and MDA-IBC-3 cell lines were seeded at moderate density overnight. Two hours before transduction, the medium was changed, and then transductions were carried out for 24 h in the presence of 8 µg/mL polybrene (Sigma). The cells were then FACS sorted and further expanded for injections. The MSC were labeled similarly but with a red fluorescent protein (RFP) generously obtained from Tsien's lab (UCSD) that was cloned into pFUGW construct in place of GFP.

### Mammosphere assay

To generate mammospheres, cells were grown in serum-free, growth factor enriched conditions in low attachment plates [4], [12], [13]. Cells were grown in 6-well ultra-low attachment plates in serum-free MEM supplemented with 20 ng/ml bFGF, 20 ng/ml EGF and B27 (all from Invitrogen). Cells were plated at 20,000 cells/ml unless specified otherwise. Suspension cultures were incubated for 1–10 days. Colonies were counted with an automated colony counter (Oxford Optronix, Oxford, UK) following MTT assay in order to increase the contrast and allow automatic detection of spheres.

### MSC conditioned media

MSC spheroids were generated by plating MSC at 20,000 cells/ml in low attachment plates and cultured in mammosphere serum-free growth factor enriched media in conditions identical to mammosphere growth [4]. MSC spheroids were cultured for 5 days in suspension cultures. Spheres were then removed by centrifugation and supernatant was used as MSC conditioned media.

### Western Blot

Mammospheres grown as described above were incubated for 5 days in 25% (volume) conditioned media derived from MSC spheroids. Spheres were collected, resuspended in 1x RIPA lysis buffer and western blots were performed according to standard protocols. Briefly, aliquots of the supernatants containing 40 µg protein were electrophoresed on 4–20% gradient sodium dodecyl sulfate-polyacrylamide gels (Invitrogen) and transferred to polyvinylidene fluoride membranes (Bio-Rad Laboratories). The membranes were incubated in 5% nonfat milk for 1 hour at room temperature and then incubated at 4°C overnight with the following antibodies: E-cadherin, N-cadherin, vimentin, fibronectin (BD Biosciences), tenascin C (Santa Cruz), rhoC (Abcam) and β-actin (Sigma). After incubation with the secondary antibody, the membranes were washed and immunoreactivity was detected by enhanced chemiluminescence. β-actin was used as a loading control.

### *In vivo* studies

Ten week old NOD/SCID gamma null mice (The Jackson Laboratory, USA) were housed and used in accordance with institutional guidelines of the University of Texas, M.D Anderson Cancer Center under the Institutional Animal Care and Use Committee (IACUC) approved protocols (ACUF 07-08-07213). The UTMDACC's animal care and use program has been fully accredited by the Association for the Assessment and Accreditation of Laboratory Animal Care International (AAALAC). GFP^+^ MDA-IBC-3 and MCF-7 cells were injected with 0, 5 and 10% MSC subcutaneously on both hindlimb of mice (5 mouse/group). A total number of 1×10^6^ cells in 100 µl of PBS were administered per injection site. Mice injected with MCF-7 tumors were simultaneously implanted in the nape of the neck with 17β-Estradiol pellets (0.36 mg/pellet, 60 days release (Innovative Research). Tumor growth was monitored with caliper measurements. When tumors were approximately 1.0 cm in size, mice were euthanized and tumors were excised. A portion of tumor was formalin fixed, paraffin-embedded, sectioned, and stained with immunohistochemistry to detect E-cadherin. An additional portion of tumors were mechanically disrupted, digested with collagenase (12.5 mg/ml, 2 hours) and filtered through a 40 µm filter (BD Biosciences). Tumor derived cells were then subjected to cell sorting using flow cytometry to isolate GFP^+^ cells. GFP^+^ cells were then centrifuged and resuspended in PBS and used for mammosphere formation and western blot analysis. Three thousand GFP^+^ tumor derived cells were plated into low attachment 96 cell plates in mammosphere media [3]. The number of mammospheres in each well was counted after 15 days after seeding. Remaining GFP^+^ xenograft derived cells were pelleted and resuspended in lysis buffer for western blot analysis.

## Results

### MSC promote mammosphere formation in normal mammary epithelial cells

**Figure 1:**
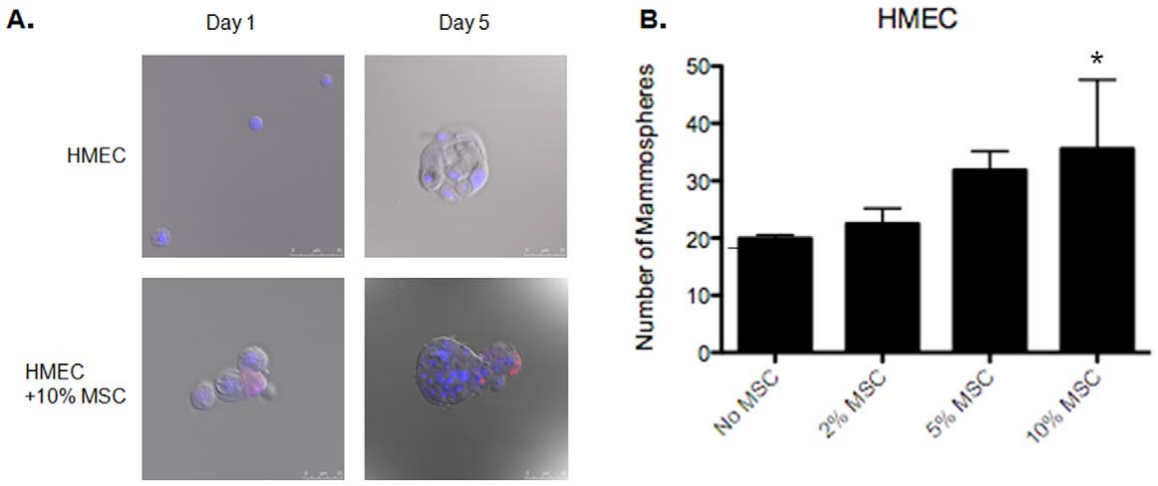
MSC increase mammosphere formation in normal mammary epithelial cells. HMEC and RFP^+^ MSC were co-plated in low-density suspension cultures. MSC associated with developing HMEC spheres on days 1–5 after plating (A). The number of mammospheres formed increased with increasing number of MSC plated (* No MSC vs. 10% MSC, p<0.001) (B).

Normal human mammary epithelial cells (HMEC) with or without 2, 5 or 10% MSC labeled with red fluorescent protein (RFP) were plated into low-density suspension cultures in serum-free growth factor supplemented media into ultra-low attachment plates. Twenty-thousand cells were plated per well, including MSC. MSC were found to interact as spheres and as single cells with HMEC as soon as one day after plating (Figure 1A). After 5 to 10 days, MSC identified as RFP positive (RFP^+^) cells were associated with HMEC mammospheres and remained as part of spheres for up to 15 days (Figure 1B). HMEC cells plated with MSC demonstrated a dose-dependent increase in mammosphere formation, with a nearly two-fold increase in sphere formation in the presence of 10% MSC (Figure 1B, p<0.01).

### MSC promote mammosphere formation in human breast cancer cell lines

**Figure 2:**
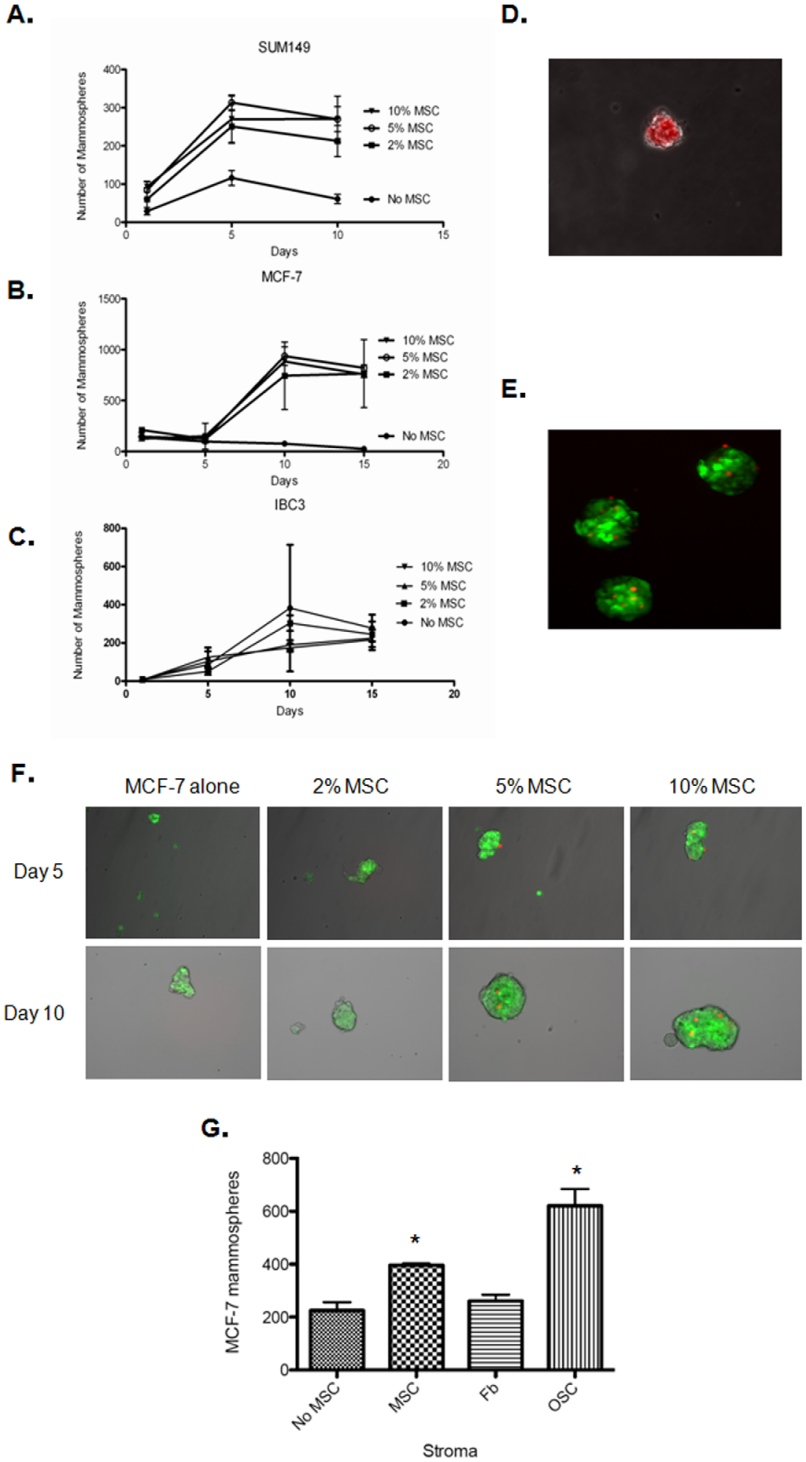
MSC increase mammosphere formation in breast cancer stem cells. Number of mammospheres formed/40,000 cells plated in low density suspension cultures increased with an increasing percentage of MSC (*p<0.001 as compared to no MSC control) for breast cancer cell lines SUM149 (A), MCF-7 (B) and MDA-IBC-3 (C). MSC formed spheroids under mammosphere culture conditions (D). Low-magnification image of MCF-7 (GFP^+^) mammospheres demonstrating integrated red-fluorescent protein expressing MSC (E). MCF-7 mammospheres 5 and 10 days after plating with and without co-plated MSC (0, 2, 5 and 10% MSC) (F). MCF-7 mammosphere formation is shown one day after plating with 10% stroma cells; MSC (human bone marrow-derived MSC), Fb (human CD16 normal human lung fibroblasts) and OSC (human omental derived adipocyte stem cells) (G) (*p<0.05 as compared to no MSC control).

Next, we investigated whether direct exposure to MSC would influence mammosphere formation of tumorigenic breast cell lines. MCF-7 cells (labeled with green fluorescent protein, GFP^+^) were plated with or without increasing percentages of MSC (RFP^+^ cells) at low-density into low-attachment plates with growth factor supplemented serum-free media. MCF-7 is an estrogen receptor-positive breast cancer, while SUM149 and MDA-IBC-3 are both derived from a hormone receptor-negative inflammatory breast cancer cells. Her2-neu is overexpressed in MDA-IBC-3 cells. The presence of 2% MSC cells markedly increased the number of SUM149 mammospheres formed (60 vs. 270, p<0.01) (Figure 2A). MCF-7 mammospheres similarly increased markedly on exposure to MSC (77 vs. 884, p<0.01 at 10 days after plating) (Figure 2B). No statistically significant difference was observed between 2%, 5% and 10% MSC. The presence of MSC did not significantly increase mammospheres in MDA-IBC-3 cells (Figure 2C). The MSC containing mammospheres were spherical and well organized in comparison to spheres formed from MCF-7 cells alone at this time point (Figure 2F). Sphere formation was readily assessable within several days.

MSC grew readily as spheres in mammosphere culture (Figure 2D), but incorporate into spheres from tumor cells as single cells (Figure 2E). Mammosphere size was significantly increased (108 vs. 189 µm in spheres composed of MCF-7 cells alone as compared to spheres plated with 20% MSC, p = 0.003) a finding not accounted for simply by increased incorporation of MSC into the spheres, Figure 2E and 2F. The total number of cells plated was held constant in each experiment such that fewer tumor cells were plated in the combined cultures.

To determine if this quality is unique to MSC, we compared mammosphere in the presence of 5% CD16 fibroblasts, which are derived from human embryonic lung, and adipose stromal cells derived from human omentum (OSC). Mammosphere formation was increased in the presence of MSC and OSC and not significantly in the presence of differentiated fibroblasts (Figure 2G).

### MSC-conditioned media increases mammosphere formation

**Figure 3:**
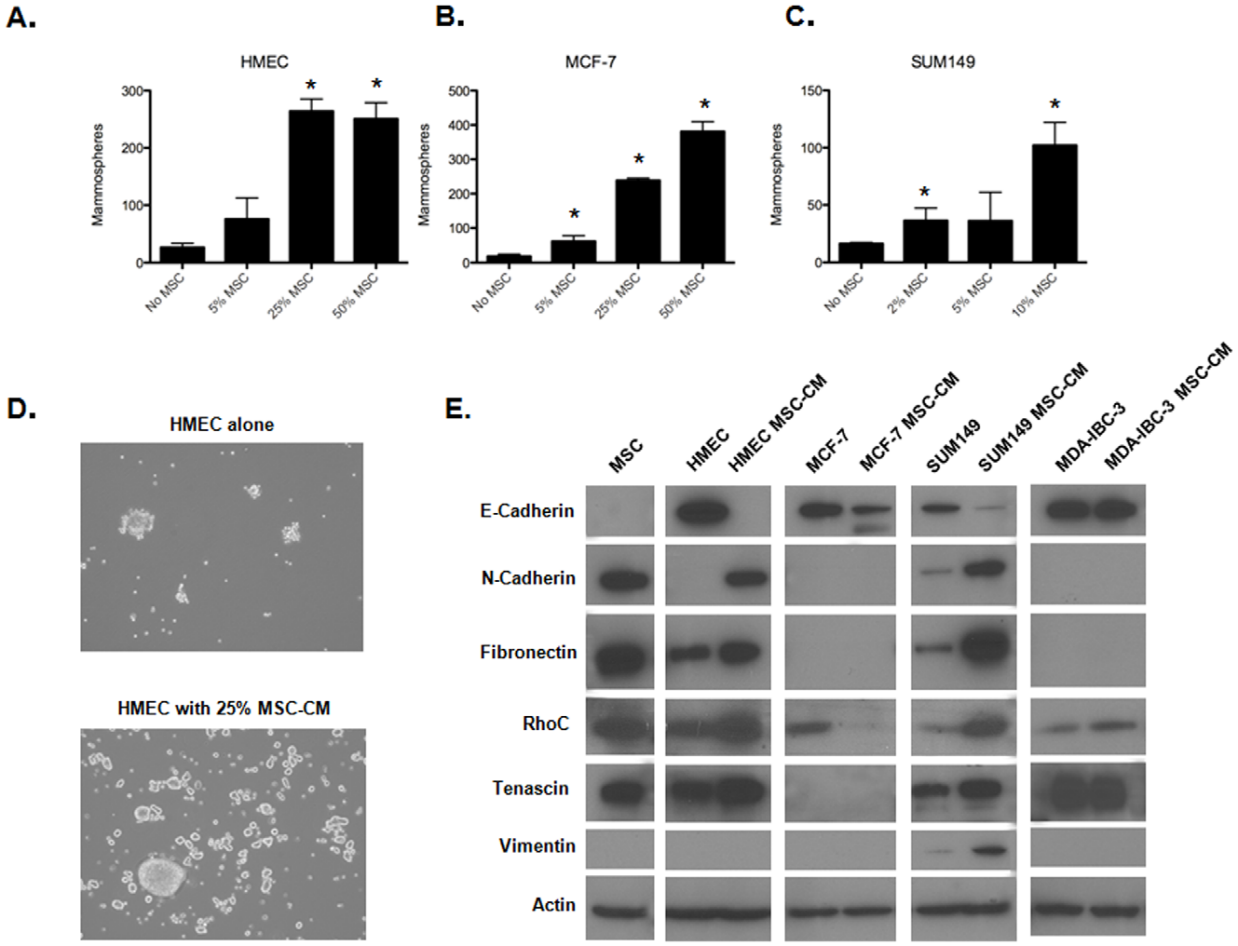
Impact of MSC conditioned media on mammosphere formation of normal human mammary epithelial cells and breast cancer cells. Number of HMEC (A), MCF-7 (B) and SUM149 (C) spheres were counted one day after plating in the presence of an increasing percentage of volume of MSC-CM. Images of 3D HMEC suspension cultures with and without MSC-CM (D) (*p<0.05 as compared to no MSC control). Western blot analysis of HMEC, MCF-7, SUM149 and MDA-IBC-3 spheres samples cultured with or without MSC-CM (E).

To determine if direct cell contact between MSC and tumor cells was required, the impact of MSC conditioned media (MSC-CM) on mammosphere formation was examined. MSC-CM was generated by growing MSC as spheres using mammosphere culture conditions for 5 days (Figure 2D). HMEC and MCF-7 cells were cultured in serum-free mammosphere culture with an increasing percentage of volume of serum-free MSC-CM. A dose-dependent increase in mammosphere formation was seen in HMEC, MCF-7, and SUM-149 cells. Fifty-percent MSC-CM resulted in 9.6 and 21.0-fold increase in sphere formation after 5 days for HMEC and MCF-7 cells (Figure 3A, 3B and 3D). Ten percent volume MSC-CM resulted in a 6.4-fold increase in mammosphere formation of SUM149 cells one day after plating (p<0.01) (Figure 3C). This early time point was used to minimize any effect of sphere aggregation which increases over time even in low density culture. However, MDA-IBC-3 cells did not form more mammospheres when exposed to MSC-CM without MSC (data not shown).

To determine if mammospheres formed from MSC-CM were enriched with self-renewing cells, we passaged the spheres from MCF-7 mammospheres. MCF-7 control cells and cells grown with MSC-CM were replated as single cells. There was no difference in serial passaging of MSC-CM derived spheres and untreated MCF-7 mammospheres (data not shown).

### MSC secreted factors result in altered metastasis-related signaling molecules

Mammosphere formation in HMEC increases upon induction of the epithelial to mesenchymal transition, which is associated with cancer invasion and metastasis [14], [15]. MSC have been shown to interact with breast cancer cells in monolayer cell culture promoting epithelial-mesenchymal transition (EMT) [16]. Furthermore, MSC can increase breast cancer metastasis [15]. We thus hypothesized that MSC may modulate EMT pathways, resulting in increased mammosphere formation. To investigate this, HMEC were plated in low-density cultures with and without 25% volume of MSC-CM. After 7 days, spheres were isolated and expression of proteins involved in breast stem cell survival were examined (Figure 3E). Cell lines that exhibit increased mammosphere formation when cultured with MSC-CM all demonstrate decreased E-cadherin expression. HMEC and SUM149 cells exposed to MSC-CM additionally upregulated the pro-invasive N-cadherin protein. Snail and Slug were also found upregulated after incubation of SUM149 with MSC-CM (Supplemental Figure S2). Next, we investigated the impact of MSC-CM on expression of other mesenchymal proteins. RhoC activity has been shown to reduce EMT-induced migration and also to regulate E-cadherin expression [17] and is specifically implicated in IBC tumor progression [18]. Increased rhoC was observed in HMEC and SUM149 cells exposed to MSC-CM. While decreased E-cadherin was apparent in the luminal cell line MCF-7, N-cadherin and fibronectin are not expressed at baseline or after treatment with MSC-CM and conversely, RhoC is down-regulated in this non-IBC cell line.

MDA-IBC-3 cells which demonstrated no significant increase in mammosphere formation had no detectable decrease in E-cadherin or RhoC when cultured with MSC-CM. Fibronectin is an extracellular matrix glycoproteins which binds integrins and other cells extracellular matrix proteins which is involved in cell migration and differentiation [19]. HMEC and SUM149 derived mammospheres had higher levels of fibronectin in mammospheres exposed to MSC-CM. No fibronectin was detected in MCF-7 or MDA-IBC-3 mammospheres. Tenascin-C is expressed in the stroma of tumors and is associated with a poor prognosis [20], [21]. Tenascin-C binds to fibronectin and can reduce cell adhesion *in vitro* [22]. We found that tenascin-C was increased in mammospheres exposed to MSC-CM in HMEC and SUM149 cells. Vimentin is an intermediated filament protein which is used as a marker of the epithelial to mesenchymal transition [23]. We detected vimentin only in SUM149 cells, where it was upregulated in response to MSC-CM. Vimentin was detected in MSC after extended exposure times (data not shown).

### MSC promote growth of MDA-IBC-3 xenografts and decrease E-cadherin expression *in vivo*

**Figure 4:**
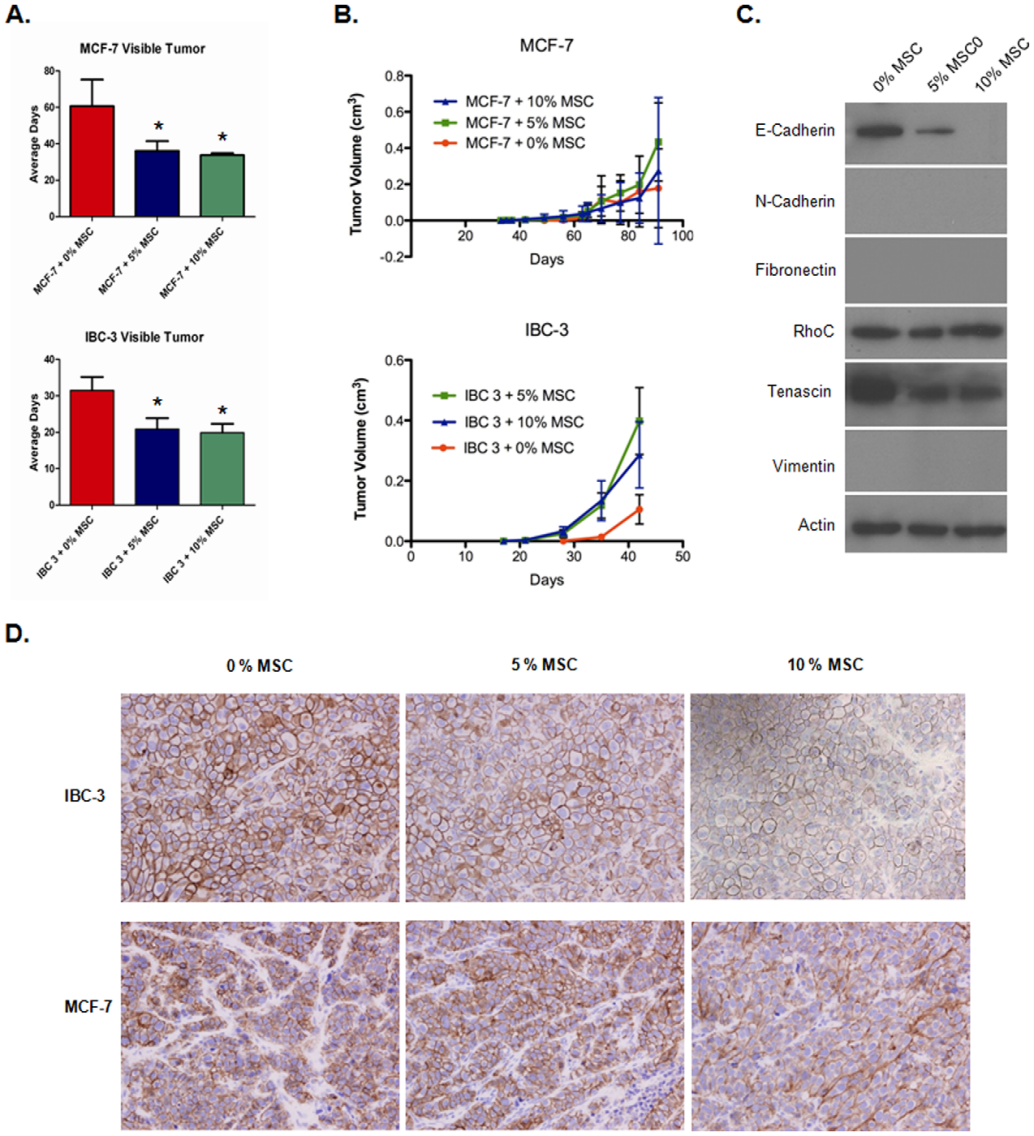
MSC promote growth of xenografts and decrease E-cadherin expression *in vivo*. GFP^+^ MDA-IBC-3 and MCF-7 cells were mixed with MSC prior to injection into the hindimb of NOD-SCID mice. Tumor growth was measured with twice weekly caliper measurements to determine the average days to develop detectable tumors (A) and tumor growth (B) (*p<0.05 as compared to no MSC control). Western blot analysis was performed on GFP^+^ MDA-IBC-3 cells from xenografts with and without co-injected MSC (C). Immunohistochemistry images of E-cadherin in tumor sections from MDA-IBC-3 and MCF-7 xenografts with and without co-injected MSC (D).

To determine if MSC impacted the TIC-enriched mammosphere forming cells *in vivo*, MCF-7 and MDA-IBC-3 cells were injected into NOD/SCID gamma null mice with and without MSC. Consistent with previous reports [15], [24], MCF-7 tumor growth was not significantly increased by co-injection of MSC (Figure 4A). However, MCF-7 tumors were detected earlier when tumor cells were co-injected with MSC (average of 34 vs. 61 days after injection with 10% MSC, p<0.001). MDA-IBC-3 tumors co-injected with MSC were detected earlier and also grew more rapidly (average of 20 vs. 32 days after injection with 10% MSC, p<0.05 Figure 4A and 4B).

To examine the *in vivo* effect of MSC on TIC surrogates, we examined mammosphere formation and cell surface marker expression from tumors injected with and without MSC. To accomplish this, GFP expressing tumor cells were isolated from both xenograft tumors injected with and without MSC (0, 5 and 10% MSC). Average percentage of GFP^+^ cells isolated from MDA-IBC-3 and MCF-7 tumors was 87% (standard deviation 9.5%) and 57% (standard deviation 25%) respectively. No mammospheres were formed from GFP^+^ MCF-7 tumor cells. Mammosphere formation efficiency from GFP^+^ MDA-IBC-3 xenograft cells was 3.3%, but was not significant different in tumors with co-injected MSC (data not shown). Co-injection with MSC did not increase the percentage of CD44^+^CD24^−^ MDA-IBC-3 cells. The percentage of GFP^+^CD44^+^CD24^−^ cells in xenografts generated with 0%, 5%, and 10% MSC was 1.75%, 1.02%, and 0.45% respectively (p = NS). Because MCF-7 tumors were smaller and had fewer GFP^+^ cells, there were insufficient cells remaining after sorting for mammospheres to assess GFP^+^CD44^+^CD24^−^ cells in MCF-7 cells. *In vitro* MSC-CM did not increase considerably the CD44^+^CD24^−^ population on SUM149 cells (Supplemental Figure S3). Immunohistochemical assessment of Ki-67 (2 tumors per group) demonstrates staining in 60–95% of xenograft cells (from MCF-7 and MDA-IBC-3 cells) with no significant difference or trend between groups. To determine if MSC altered cadherin expression *in vivo*, GFP^+^ MDA-IBC-3 tumor cells were isolated from xenografts with and without MSC were isolated and western blots were performed (Figure 4C). E-cadherin expression was decreased in MDA-IBC-3 tumor cells with co-injected MSC (Figure 4C). RhoC expression in MDA-IBC-3 xenografts was similar with and without co-injected MSC. MDA-IBC-3 did not express detectable N-cadherin and fibronectin and vimentin. Tenascin-C expression was lower in tumors with co-injected MSC. Insufficient protein was available for western blot analysis of E-cadherin *in vivo* from GFP^+^ cells derived from MCF-7 tumors. Therefore, immunohistochemistry was performed on MCF-7 xenografts as well as MDA-IBC-3 xenografts to detect E-cadherin protein. Decreased E-cadherin expression was appreciated in the presence of 5 and 10% MSC in MDA-IBC-3 tumors, consistent with western blot analysis (Figure 4C and D). In MCF-7 tumors, staining patterns were heterogeneous, with areas of lower E-cadherin expression were identified in tumors containing MSC and were absent in tumors without co-injected MSC (Figure 4D).

## Discussion

The discovery that MSC demonstrate a unique tropism for the tumor microenvironment has led to a great deal of interest in understanding the function of MSC within tumors [9], [25], [26]. MSC have been shown to increase the growth of certain cancers when injected together with MSC and can increase the incidence of breast xenograft metastasis [15]. However, a great deal remains unknown about the interaction of MSC and breast cancers. In this study, we report that MSC increase the capacity of normal mammary epithelial cells and established breast cancer cell lines (MCF-7 and SUM149) but not short-term primary breast cells (MDA-IBC-3) to form primary mammospheres. Both direct exposure to MSC, as well as MSC-conditioned media, promoted mammosphere formation from HMEC, MCF7, and SUM149 demonstrating that MSC secrete a sphere-promoting factor to which that these lines are sensitive. We hypothesize that this may occur through an increase in EMT in breast cancer cells, since MSC provoke a cadherin switch, characteristic of EMT, in normal and malignant (SUM149) breast mammospheres. Decreased full-length E-cadherin expression was observed *in vivo* or *in vitro* in all lines examined. In cells that express N-cadherin at baseline, this downregulation of E-cadherin was accompanied by an increase in mesenchymal proteins including N-cadherin and fibronectin.

The mesenchymal phenotype has previously been reported to increase primary mammosphere formation. Mani *et. al.* demonstrated that expression of EMT regulating transcription factors, *snail* and *twist*, increased mammosphere formation [14]. Furthermore, mammospheres from *snail* and *twist* over-expressing cells contained a higher percentage of cells with stem cells markers suggesting that EMT generates cells with stem cells properties [14]. Our findings are consistent with previous studies in which MSC have been shown to interact with breast cancer cells in monolayer cell culture to promote epithelial-mesenchymal transition (EMT) [16].

We examined whether exposure to MSC-CM could be enriching for the growth of cells with stem cell properties. However, while primary sphere formation was reproducibly increased in a dose dependent manner in established cell lines, secondary sphere formation was not increased and neither sphere formation nor CD44^+^CD24^−^ cells were increased in MDA-IBC-3 tumors cultured with MSC. This suggests that the expansion of mammospheres may represent an amplification of non-self-renewing progenitors involved in the observed tumor growth promotion and increased invasion [15] rather than self-renewing tumor stem cells. Rigorous *in vivo* serial passaging experiments will be required to determine if MSC influence the cancer stem cell population.

Dittmer *et. al.* recently reported that MSC can integrate into formed breast cancer spheroids, altering E-cadherin expression and increasing breast cancer cell invasion. In contrast to our work, these authors observed that MSC containing aggregates were more disorganized, while we have observed that spheroids with MSC appear more spherical and well organized than non-MSC containing spheres [27]. This difference may be explained by the fact that Dittmer *et. al.* were examining the effects of MSC added to aggregates of spontaneously floating cells in monolayer serum-containing culture as compared to our study in which the effect of MSC was evaluated in spheres grown from single cells plated at clonal density in stem cell promoting serum-free media. The process of MSC integration into established spheres may result in disrupted morphology. This may imply a differential effect on TIC secondary to less reliance on E-cadherin for adhesion in TIC.

Strikingly, while the down-regulation of E-cadherin was observed *in vitro* or *in vivo* in all cells examined, there are distinct differences across cell lines in expression of other pro-invasion and mesenchymal proteins. E-cadherin was decreased in both the estrogen receptor (ER) positive luminal E-cadherin positive cells (MCF-7) and in the ER negative E-cadherin positive cells (SUM149). E-cadherin is a cell adhesion protein which functions as a tumor supressor gene with forced expression of E-cadherin resulting in reduced tumor cell invasion [28]. High levels of E-cadherin are associated with better clinical outcomes in patients with non-IBC [29]. However, E-cadherin has been proposed to have a unique role in IBC where E-cadherin expression is present in both the primary and in the tumor emboli found in the angiolymphatics in the breast [18], prompting the hypothesis that E-cadherin expression is dynamic, and potentially only transiently down-regulated during metastasis [30]. In the Mary-X IBC mouse model, it has been shown that the aggregates of tumor in emboli are facilitated by a functional E-cadherin/β-catenin axis [31] and that knock-down inhibits aggregates, and that further these aggregates metastasize as E-cadherin positive clusters through a passive process rather than hemotogenous spread [31]. To that end, dominant negative E-cadherin in SUM149 cells inhibits invasion as expected in non-IBC tumors. Here, however, we demonstrate for the first time that MSC can promote the growth of an IBC cell line, MDA-IBC-3, and that E-cadherin is down-regulated in the larger MSC co-injected MDA-IBC-3 xenograft. Given that IBC clearly can develop metastatic disease via hematogenous spread as well as potentially passive spread via the angiolymphatic channels we propose that IBC cells are capable of both E-cadherin positive non-hemotogenous spread as well as more well-described E-cadherin negative promoting invasive behavior. We can not determine from this model if E-cadherin is re-expressed after metastases are established.

Our findings demonstrate that MSC provoke breast cancer cells to form mammospheres and assume a more mesenchymal phenotype and that MSC integrate into breast cancer mammospheres and decrease E-cadherin expression in both ER positive luminal E-cadherin positive cells and ER negative E-cadherin positive IBC cells. This suggests that MSC may represent a novel therapeutic target either independently or by inhibiting the effects of MSC on cadherin expression in breast cancer cells.

## Supporting Information

Table S1 STR data from xenografts and *in vitro* culture of MDA-IBC-3 cell line.
(0.02 MB PDF)

Figure S1 MSC express characteristic cell surface markers and demonstrate multi-lineage potential. Cell surface marker expression was characterized with flow cytometry (A). MSC were subjected to differentiation assays with and stained with Alizarin Red S to detect osteoblast differentiation, Oil Red O to detect adipocyte differentiation and Alcian Blue to detect chondrocyte differentiation.
(2.05 MB TIF)

Figure S2 Western blot analysis of SUM149, MDA-IBC-3, MCF-7, and HMEC spheres samples cultured with or without MSC-CM.
(0.57 MB TIF)

Figure S3 Flow cytometry analysis of expression of CD24 and CD44 on SUM149 cells cultured as spheres with and without MSC-CM.
(2.26 MB TIF)
