# Supplementary material for: Mesenchymal Stem Cells Promote Mammosphere Formation and Decrease E-Cadherin in Normal and Malignant Breast Cells
Source: PLoS One. 2010 Aug 16;5(8):e12180. doi: 10.1371/journal.pone.0012180 (PMC2922340; doi:10.1371/journal.pone.0012180)
Supplement: Table S1 — STR data from xenografts and in vitro culture of MDA-IBC-3 cell line. (0.02 MB PDF) [file pone.0012180.s001.pdf]

DNA Profile (STR) Using Promega's Cell ID System	Amelogenin: X<br>CSF1PO: 12,13<br>D13S317:11,12<br>D16S539: 9,12<br>D5S818: 10,11<br>D7S820: 8,12<br>THO1: 8<br>TPOX: 10,11<br>vWA: 18,19

Short tandem repeat (STR) amplification and fragment analysis. STRs were amplified using the Cell ID system (Promega, Madison, WI). Briefly, 2 ng of genomic DNA from each sample was amplified using Taq Polymerase and optimized primers for the following ten loci: D21S11, D5S818, D13S317, D7S820, D16S539, CSF1PO, TPOX, TH01, Amelogenin and vWA. PCR products were run in an optimized thermocycler setting and analyzed using capillary electrophoresis in the DNA Core Lab at MD Anderson. The ABI prism 3100 Genetic Analyzer with GeneMapper ID 4.0 Software was used to analyze the amplified STR fragments to determine fragment length and the number of repeats in each fragment. DNA from K562 cells and reactions without DNA served as positive and negative control, respectively.

Results. The STR data from xenografts and *in vitro* culture of MDA-IBC-3 were the same and is shown in Table S1. The cell line is unique in that no match was found with existing STR cell line databases. Reproducible results were demonstrated 12 months apart.
