# Supplementary figures and images for: Mesenchymal Stem Cells Promote Mammosphere Formation and Decrease E-Cadherin in Normal and Malignant Breast Cells
Source: PLoS One. 2010 Aug 16;5(8):e12180. doi: 10.1371/journal.pone.0012180 (PMC2922340; doi:10.1371/journal.pone.0012180)

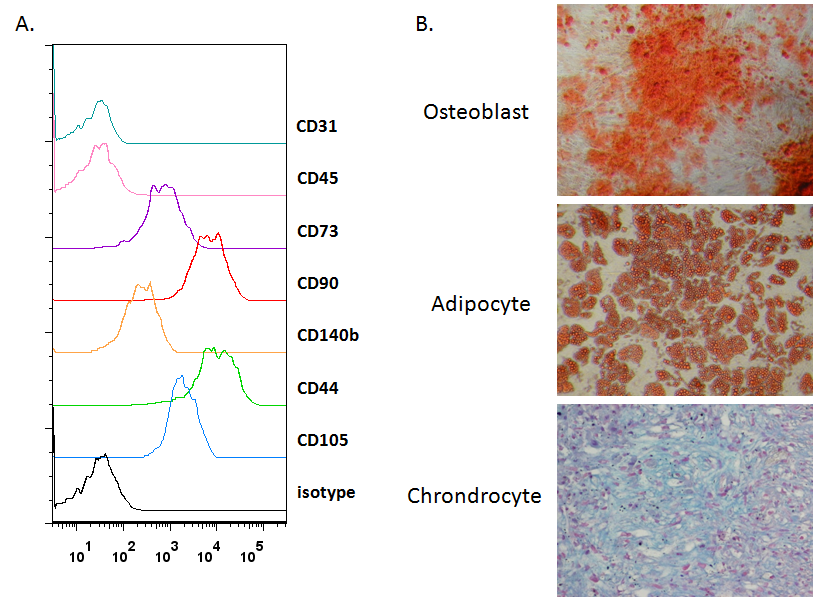

Supplement: Figure S1 — MSC express characteristic cell surface markers and demonstrate multi-lineage potential. Cell surface marker expression was characterized with flow cytometry (A). MSC were subjected to differentiation assays with and stained with Alizarin Red S to detect osteoblast differentiation, Oil Red O to detect adipocyte differentiation and Alcian Blue to detect chondrocyte differentiation. (2.05 MB TIF) [file pone.0012180.s002.tif]

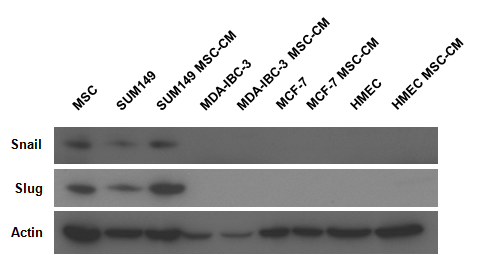

Supplement: Figure S2 — Western blot analysis of SUM149, MDA-IBC-3, MCF-7, and HMEC spheres samples cultured with or without MSC-CM. (0.57 MB TIF) [file pone.0012180.s003.tif]

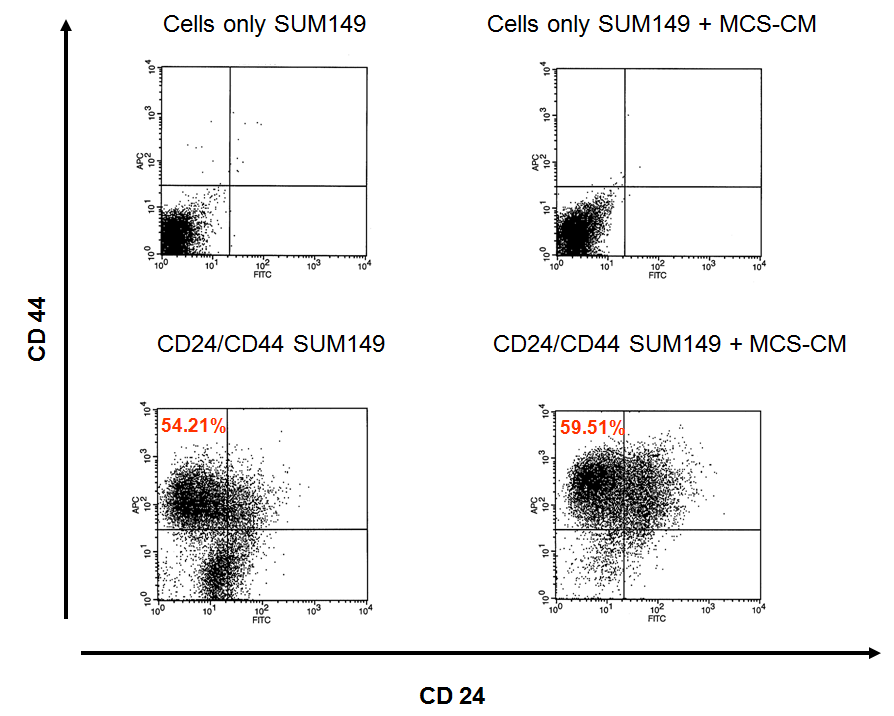

Supplement: Figure S3 — Flow cytometry analysis of expression of CD24 and CD44 on SUM149 cells cultured as spheres with and without MSC-CM. (2.26 MB TIF) [file pone.0012180.s004.tif]
